# Transplantation of fecal filtrate to neonatal pigs reduces post-weaning diarrhea: A pilot study

**DOI:** 10.3389/fvets.2023.1110128

**Published:** 2023-03-16

**Authors:** Christina Larsen, Amanda B. Andersen, Helena Sato, Anders Brunse, Thomas Thymann

**Affiliations:** Department of Veterinary and Animal Science, University of Copenhagen, Frederiksberg, Denmark

**Keywords:** fecal filtrate transplantation, post-weaning diarrhea, gut microbiome, neonatal, mucosa

## Abstract

Post-weaning diarrhea (PWD) remains a major source of mortality and morbidity in swine production. Transplantation of bacteria-free filtrate of feces (fecal filtrate transplant, FFT) has shown gut protective effects in neonatal pigs, and early postnatal establishment of the gut microbiome is suggested to determine later stability and robustness of the gut. We, therefore, hypothesized that early postnatal transplantation of bacteria-free feces would have a protective effect against PWD. Using fecal filtrates derived from healthy lactating sows, we compared oral administration of fecal filtrate transplantation (FFT, n = 20) and saline (CON, n = 18) in newborn piglets. We assessed growth, diarrhea prevalence, blood parameters, organ measurements, morphology, and gut brush border enzymes and analyzed luminal bacterial composition using 16S rRNA gene amplicon sequencing. The two groups showed similar average daily gain (ADG) during the suckling period, whereas in the post-weaning period, a negative ADG was observed in both groups. While diarrhea was largely absent in both groups before weaning, there was a lower diarrhea prevalence on days 27 (*p* = 2.07^*^10^−9^), 28 (*p* = 0.04), and 35 (*p* = 0.04) in the FFT group relative to CON. At weaning on day 27, the FFT group had higher numbers of red blood cells, monocytes, and lymphocytes, while on day 35, i.e., 1 week after weaning, the two groups were similar regarding hematology. The biochemical profile was largely similar between FFT and CON on days 27 and 35, except for a higher level of alanine aminotransferase and a lower level of Mg in the FFT group. Likewise, organ weights relative to body weight were largely similar on day 35, albeit with a lower stomach weight and more colon content in FFT relative to CON. Gut mucosal percentage and mucosal enzyme activity were similar between the two groups on days 27 and 35. Gut bacterial composition was slightly different on day 35 but not on day 27. In conclusion, early postnatal administration of FFT, showed positive clinical effects in post-weaning pigs, albeit with subtle effects on the gut mucosa and microbiome. Prophylactic treatment with FFT may offer a means to reduce morbidity, yet larger studies are required to document effect size.

## 1. Introduction

Post-weaning diarrhea (PWD) is one of the most frequent gut diseases in commercial pig production. It increases mortality and morbidity rates substantially (1), and associates with high antibiotic use and consequently increased anti-microbial resistance (2). Alternative rearing strategies to improve gut health in newly weaned piglets have been investigated through many years, and relate mainly to weaning age, creep feeding, diet composition, housing conditions, genotypes, and pro- and prebiotics (3–6). Common to these factors is their influence on the gut microbiome during the critical transition from sows' milk to solid feed, where dysbiosis and overgrowth of enterotoxigenic *Escherichia coli* (ETEC) is commonly seen (3, 5). Whereas pigs are largely devoid of microorganisms at the time of birth, they are rapidly colonized after birth when exposed to maternal feces, maternal skin, and environmental microorganisms. The gut microbiota fluctuates substantially in the early postnatal phase (7) and continues to evolve until adulthood (8). The microbiome is of importance for the host metabolism and health (9), and early postnatal colonization may be an important factor for gut health, both short and long-term (9). The evolvement of microbial colonization helps establish the gut barrier function as well as maturation of the immune system (10). On that note, a high abundance of *Prevotellaceae, Lachnospiraceae, Ruminococaeae*, and *Lactobacilaceae* and a decrease of *Fusobacteriaceae* and *Corynebacteriaceae* on day seven of life, has been shown to associate with a lower occurrence of PWD, whereas a high abundance of *Enterobacteriaceae* and low abundance of Bateroidetes has been shown to associate with increased PWD (9). During the weaning process, the microbial diversity becomes reduced and is often associated with a low abundance of *Lactobacillus*, and a high abundance of *Clostridium spp., Prevotella spp*., and *Proteobacteria* (11). The rapid development in microbial composition in early life raises the question of whether this developmental trajectory can be influenced to secure better gut health. Several reports indicate that microbial interventions in the early postnatal phase represent a critical window to improve gut health and optimize immunity and growth traits (12, 13). While interventions that relate to microbial changes in early life relate mainly to antimicrobials or pre-and probiotics, other strategies such as fecal microbiota transplantation (FMT) have also gained interest in recent years (14). Studies on use of FMT in pigs have shown a positive effect on growth performance, gut health, and a decrease in PWD (13, 15–20). Some studies indicate that the positive effect of FMT associates with an increase in diversity of the microbiome, and a high abundance of Firmicutes, *Bacteroides*, and *Prevotella* (19, 21). This observation is, however, not consistent across studies, and may relate to inappropriate donor-recipient matches, which may involve age and breed (18, 21, 22). The FMT intervention uses stool from a healthy donor that is transplanted into the recipient's intestinal tract to modulate the microbiota (23). Despite the reported positive effects of FMT, there is also an inherent risk of inducing infections with viral or bacterial pathogens, particularly in newborn pigs that are not yet fully immunized with sows' colostrum. Accordingly, we have shown that FMT provided orally to hyperimmunized neonatal pigs, induces infection and sepsis-like conditions (24). The infections likely occur in the small intestine as rectal application appeared to be both safe and had a positive clinical effect on the host. Rectal application of fecal transplants may however not be feasible under practical farming conditions. This creates an incentive to improve the method for oral application to make it a safe and efficient therapy.

In search of better microbial therapies, it was recently shown that bacteria-free filtrate transplantation has a promising safety and efficacy profile in a piglet model of gut inflammation (25). Filtration removes bacteria, fungi, and parasites, leaving only viruses specific to eukaryotic cells and bacteriophages (collectively referred to as the virome), i.e., partly eliminating the risk of transferring pathogenic microorganisms (bacteria, fungi, protozoa) while maintaining beneficial effects of the remaining bacteriophages (25, 26), although the risk of transferring pathogenic viruses persists. Bacteriophages are highly host-specific viruses that target only bacteria and cause cell lysis by the reproductive life cycle, i.e., lytic cycle (27). Bacteriophages are omnipresent across bacterial ecosystems including the mammalian gut and therefore the piglet gut as well (25–27). During early gut colonization, bacteria and bacteriophages dynamically interact with one another, resulting in compositional changes over time (28). The bacteriolytic nature of bacteriophages constitutes a prevention method targeting bacterial infections in the piglet gastrointestinal tract as an alternative strategy to antibiotic treatment (29). An effect of the bacteriophage-enriched dietary supplement, with phages isolated from pig manure, was recently reported as a therapy treatment against PWD involving ETEC infections in weaned pigs (30). The results indicate an effective outcome of alleviation of acute O149 E. coli F4 (K88) fimbriae (ETEC F4) infection with the use of bacteriophages in pigs in the post-weaning period (30). The effects of bacteriophage therapy treatment included several physiological factors such as ratio between villous height and crypt depth in duodenum and jejunum, but also fecal consistency and the adhesion score of ETEC in ileum and caecum (30).

From the notion that the immediate postnatal period may be a window of opportunity for transplantation, we hypothesized that early postnatal transplantation of bacteria-free feces would improve growth and survival rates, and reduce PWD by modulation of the gut microbiome. This was designed as a pilot study to provide evidence for a larger follow-up study.

## 2. Materials and methods

The experiment was approved by the Danish Animal Experimentation Inspectorate, license number 2020-15-0201-00520.

### 2.1. Animals housing and experimental design

Thirty-eight vaginally born, term piglets [Danbred (Duroc × Danish Landrace × Yorkshire)] were selected from 19 sows of parity 1–6 on a Danish conventional herd (Burkal, Denmark). On day one, two piglets from each litter were selected and ear-tagged. Selection criteria were that the average body weight should be close to average body weight of the litter, and with equal sex distribution. The selected pigs remained with their own mother during the entire lactation period and were assigned to either a control group (CON; n=18) or a treatment group with fecal filtrate transplantation (FFT; n=20). Both groups had equal average body weights on day one of life. All piglets had free access to water and a creep area with a heating lamp. On day two to three of life, the piglets received an oral suspension of Baycoxine Vet^®^ (50 mg/mL, Elanco, Ballerup, Denmark) and from day two to four, the piglets received powdered iron (7 g/day, Vilofoss, Fredericia, Denmark). Antibiotics were not given prophylactically, yet if an experimental animal required treatment with antibiotics, it was excluded from the experiment. On day 27, one pig from each litter was randomly selected for blood and tissue collection, corresponding to *n* = 6 for CON and *n* = 7 for FFT. On day 28, all remaining piglets (CON; *n* = 6, FFT; *n* = 7) were weaned into two separate pens with free access to creep feed and water. On day 35, all pigs were euthanized to collect blood and tissue samples.

### 2.2. Experimental feed

All piglets received milk from their sow until weaning, and from days two to five, the piglets received a yogurt mix in a creep feeder (BabySuin, NutriSuin, Hapert, Holland) (Table 1). From days six to 27, they received a plant-based wet feed in a creep feeder (PreSuin, NutriSuin) (Table 2) twice per day. At weaning the piglets received solid feed semi *ad libitum* (weaners mix from 6 to 9 kg, Vilofoss, Fredericia, Denmark) (Table 3). All diets were free of antibiotics and pharmaceutical zinc oxide.

**Table 1:** Nutrient composition of BabySuin, NutriSuin^®^.

**Calculated analysis**	**%/kg**
Crude protein (%)	19.0
Crude fat (%)	15.5
Ash (%)	5.5
Lysine	1.4
Methionine	0.5

All piglets received BabySuin from days two to five of life.

**Table 2:** Nutrient composition of PreSuin pro, NutriSuin ^®^.

**Calculated analysis**	**%/kg**
Crude protein (%)	16.9
Crude fat (%)	11.4
Ash (%)	4.6
Lysine	0.76
Methionine	0.26

All piglets received PreSuin from days seven to eight of life.

**Table 3:** Nutrient composition of weaner diet.

**Ingredients**	**%/kg**
75%Wheat/25%barley	45.6
Wheat	26.0
TripleA 68 AX3 digest GMO	10.9
Fat	2.5
Landmix1 6 62–346 conc. 500 kg 6–9kg	14.9
**Calculated analysis, %**	
Metabolized energy, MJ/kg	13.8
Crude protein	17.3
Crude fat	4.93
Lysine	11.9
Methionine	3.94

All piglets received solid feed from days 28–35.

### 2.3. Donor selection, screening, and preparation

Fresh maternal donor material was collected from three healthy, lactating sows of parity two to four. All donor sows were healthy with no signs of diarrhea and with no antibiotic treatments for 3 months prior to collection of donor materials. The donor material was screened for the following pathogens: rotavirus, F4- and F18-positive *E. coli, Lawsonia, Salmonella*, and *B. pilosicoli* (Kjellerup laboratory, Landbrug & Fødevarer F.m.b.A. SEGES Laboratory for Swine diseases) and only donor material free from these pathogens was pooled together and diluted at 1:6 in sterile saline. The solution was centrifuged (Hettich Zentrifugen, D-78532, Tuttlingen, Germany) in filtered falcon tubes at 5,000 × g at 4°C for 30 min. and the supernatant was filtered through a 0.45-μm syringe filter, after which it was ready to administer as bacteria-free feces, FFT. The inoculum was stored at 6°C until administration. The inoculum was administered orally with a syringe on days 1 to 6 of life. The FFT group received 6 mL of working solution per treatment, corresponding to 1 gram of original feces, and the CON group received equivalent volumes of sterile saline.

### 2.4. Clinical observations and recordings

Clinical and fecal scores were recorded on days 1–6, 14, 21, 27, and each day in the post-weaning period i.e., days 28–35. The clinical status was scored according to a clinical scoring system (1 = normal, 2 = mild symptoms, 3 = moderate symptoms, and 4 = severe symptoms). The feces score was recorded either as normal or diarrheic and daily diarrhea prevalence was calculated as the (total cases of diarrhea/total number of piglets)^*^100. The body weights were measured (Bjerringbro vægte, model no. APM-60, Bjerringbro, Denmark) on the same days as the clinical and fecal scores were assessed.

### 2.5. Post-mortem examinations and samplings

Procedures for tissue collection included anesthesia with an injection of a mix of zolazepam (25 g/ml, Virbac, Kolding, Denmark), tiletamine (25 g/ml, Virbac), ketamine (100 g/ml, MSD Animal Health, Copenhagen, Denmark), xylazine (20 mg/ml, ScanVet Animal Health A/S, Fredensborg, Denmark), and butorphanol (10 mg/ml, Biovet ApS, Fredensborg, Denmark). When full anesthesia was achieved, blood samples were drawn by cardiac puncture, and used for clinical biochemistry and hematology. The piglets were subsequently euthanized with an intra-cardiac injection of sodium-pentobarbital (400 mg/ml, ScanVet Animal Health A/S). The liver, kidneys, and spleen were excised and weighed. The stomach, small intestine, and colon were weighed before and after emptying, and their weights were calculated relative to body weight. Tissue samples and luminal content were collected from the distal small intestine, and colon (apex). Tissue was collected for assessment of morphology and gut brush border enzyme activities. Colon luminal content was collected for 16S rRNA amplicon sequencing analysis.

### 2.6. Blood analysis

Clinical hematology was measured in Ethylenediaminetetraacetic acid (EDTA)-stabilized (BD-Plymuth, PL6 7BP, UK) whole blood. Further, plasma was isolated from EDTA-stabilized blood upon centrifugation, and biochemical profile was determined using an Advia 1,800 chemistry system (Siemens Healthcare Diagnostics, Tarrytown, NY, USA).

### 2.7. Tissue analysis

Tissue of distal jejunum was fixed in paraformaldehyde, and later embedded in paraffin, and sectioned, and stained with hematoxylin and eosin for morphological evaluation. Morphological examinations of the sections were made in a blinded manner by using a standard light microscope Olympus BX41 and CellSens software (Olympus Corporation, Tokyo, Japan). Mucosal thickness relative to the thickness of the mucosa plus submucosa was measured (Fiji-ImageJ).

### 2.8. Enzymes analysis

Mucosal activity of disaccharides (maltase, sucrase, and lactase) and peptidases (aminopeptidase N, aminopeptidase A, and dipeptidyl peptidase IV) were measured in tissue homogenates as described in Sangild et al. (31).

### 2.9. Gut microbiota composition analysis

Colon luminal content was thawed, and approximately 100 mg of each sample was subjected to DNA extraction using Micro Bead beat AX kit (A&A Biotechnology, Gdańsk, Poland). 16S rRNA gene amplicon sequencing was performed using the MinION platform (Oxford Nanopore Technologies (ONT), Oxford, UK) as previously described (24). The sequencing library was prepared with a two-step PCR method targeting the V1–V8 hypervariable region of the bacterial 16S rRNA gene using previously published primer sequences and PCR reaction mix and thermal conditions (24). The resulting PCR amplicons were purified using AMPure XP beads (Beckman Coulter Genomic, CA, USA). Pooled equimolar barcoded amplicons were subjected to 1D genomic DNA by ligation protocol (SQK-LSK109) to complete library preparation for MinION sequencing. Approximately 0.2 μg of amplicons were used for the initial step of end-prep, and 40 ng of amplicon library was loaded onto an R9.4.1 flow cell. ONT data was collected, base-called, trimmed, and demultiplexed as previously described (24). The resulting fastq files were quality corrected using NanoFilt (q ≥ 10; read length >1 Kb) and subsequently subjected to taxonomic assignment against Greengenes database (13.8). Diversity and taxonomic analyses were performed using the phyloseq package in R. Alpha diversity was expressed as richness and Shannon index in data normalized to the mean read count. Bray-Curtis dissimilarity metric after cumulative sum scaling normalization was chosen for comparisons of beta diversity and analyzed by permutational multivariate analysis of variance. Analysis of differential ASVs relative abundance was carried out in DESeq2, using a probability value of 0.05 and a log-fold difference of at least two. The ggplot2 package was used to create the graphical layout.

### 2.10. Calculations and statistical analysis

Data analysis was performed using the statistical software R (version 2022.02.1 + 461, R Foundation for Statistical Computing, Vienna, Austria), and graphical illustrations were done in GraphPad Prism (version 9.3.1 (471), GraphPad Software, La Jolla CA, USA). Repeated measurements over time for continuous variables (body weight) were analyzed using linear mixed effects with models with mRI. Average daily gain, hematology, biochemistry, organ weight, gut brush border enzyme activities, and morphology were analyzed using linear models. The models were analyzed by a two-way ANOVA. Diarrhea prevalence on each day was analyzed with Fisher's exact test or Pearson's chi-square test. Models of *in vivo* data included fixed effects as treatment and sex, covariates as birth weight (diarrhea prevalence) or kill weight (hematology, biochemistry, organ weight, gut brush border enzyme activities, and morphology), and sow as a random effect (only in models for the suckling period). Model validation was done by testing normality and homoscedasticity of the residuals and fitted values. If the assumptions were not accepted, data were log-transformed to meet the criteria. Non-parametric analysis was used when data could not meet the criteria. Data are presented as means with standard deviation. *P*-values below 0.05 were regarded as statistically significant and *P*-values below 0.10 were regarded as a tendency.

## 3. Results

### 3.1. Clinical observations

**Figure 1:**
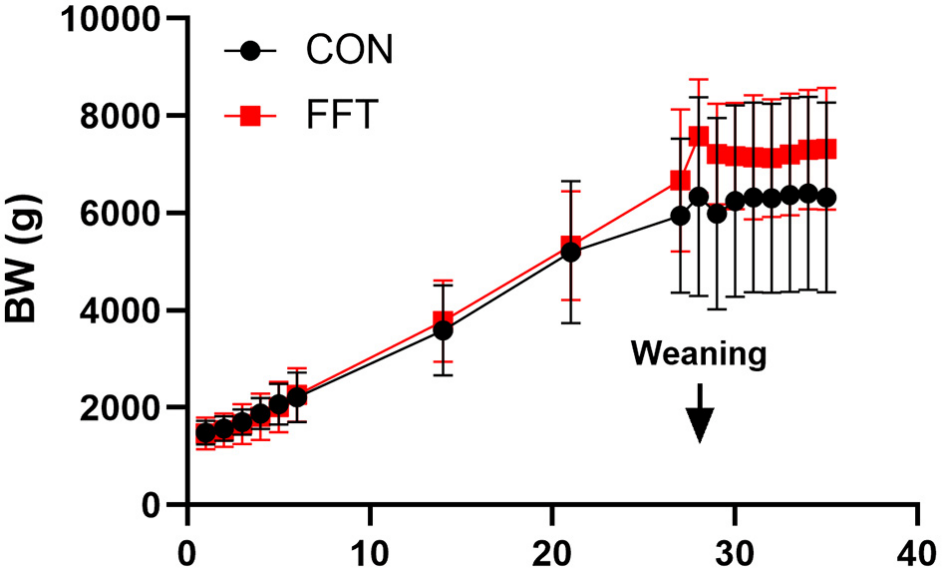
Growth curve based on daily body weights of pigs from Days 1–35. The pigs received either fecal filtrate transplantation (FFT, *n* = 7–20) or sterile saline (CON, *n* = 6–18). Data are expressed as means ± SD.

The FFT and CON group showed similar ADG during the suckling period (202 ± 46 g/d vs. 178 ± 83 g/d). Both groups showed weight loss in the post-weaning period, with FFT having the highest weight loss relative to CON (−37 ± 35 g/d vs. −2.38 ± 19.4 g/d) (Figure 1).

**Figure 2:**
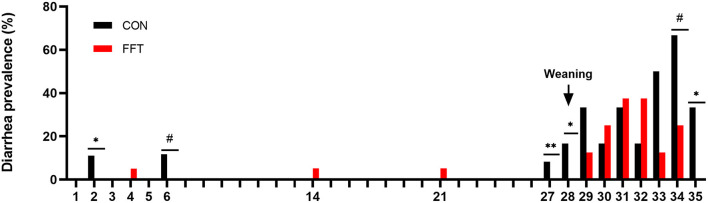
Daily diarrhea prevalence of pigs from day 1–35. The pigs received either fecal filtrate transplantation (FFT, *n* = 7–20) or sterile saline (CON, *n* = 6–18) ^#^0.05 ≤ *p* < 0.10, *p < 0.05, **p < 0.01.

Importantly, fecal filtrate transplantation reduced diarrhea prevalence significantly on days 27, 28, and 35 in the post-weaning period compared to CON. On these days the prevalence was zero in the FFT group, whereas it was 8, 17, and 33% in the CON group. On day 34, there was a tendency toward a lower diarrhea prevalence in FFT vs. CON (25 vs. 67%) (Figure 2). Diarrhea prevalence on the remaining post-weaning days was similar.

On day 27, i.e., the day before weaning, the stomach weight tended to be lower in CON compared to FFT (Table 4). After weaning, i.e., on day 35, the weight of the stomach was significantly lower in FFT compared to CON. However, the weight of the full colon was 0.64 g/kg lower in CON compared to FFT (Table 5).

**Table 4:** Relative organ dimensions of piglets euthanized at day 27 (*n* = CON: 6; FFT: 7).

**Parameter**	**Unit**	**Day**	**CON**	**FFT**	***p*-value**
Small intestine full	g/kg	27	5.09 ± 1.24	5.10 ± 0.67	NS
Small intestine empty	g/kg	27	4.06 ± 0.85	4.10 ± 0.38	NS
Stomach full	g/kg	27	2.81 ± 0.93	2.50 ± 0.64	NS
Stomach	g/kg	27	4.06 ± 0.85	4.10 ± 0.38	0.07
Colon full	g/kg	27	2.23 ± 0.48	2.48 ± 0.79	NS
Colon	g/kg	27	1.09 ± 0.19	1.19 ± 0.29	NS
Liver	g/kg	27	3.17 ± 0.34	3.35 ± 0.61	NS
Spleen	g/kg	27	0.38 ± 0.07	0.37 ± 0.11	NS
Kidney	g/kg	27	0.71 ± 0.17	0.68 ± 0.11	NS

CON, Control, FFT, Fecal filtrate transplantation. Values are expressed as mean ± SD. NS: Not significant. *Organ* dimensions are normalized to the body weight on day 27.

**Table 5:** Relative organ dimensions of piglets euthanized on day 35 (*n* = CON: 6; FFT: 8).

**Parameter**	**Unit**	**Day**	**CON**	**FFT**	***p*-value**
Small intestine full	g/kg	35	7.79 ± 2.02	6.91 ± 0.79	NS
Small intestine empty	g/kg	35	4.54 ± 0.95	4.03 ± 0.36	NS
Stomach full	g/kg	35	5.52 ± 2.42	3.39 ± 1.28	0.02^*^#
Stomach	g/kg	35	1.01 ± 0.21	0.76 ± 0.10	0.003^*^#
Colon full	g/kg	35	4.61 ± 0.54	5.25 ± 0.87	0.04^*^
Colon	g/kg	35	1.80 ± 0.24	1.90 ± 0.28	NS
Liver	g/kg	35	2.54 ± 0.76	2.69 ± 0.34	NS
Spleen	g/kg	35	0.37 ± 0.09	0.36 ± 0.10	NS
Kidney	g/kg	35	0.73 ± 0.38	0.53 ± 0.06	NS

CON, Control, FFT, Fecal filtrate transplantation. Values are expressed as mean ± SD. ^*^p < 0.05. NS: Not significant. *Organ* dimensions are normalized to the body weight on day 35. #: log-transformed data.

Relative to the CON group, FFT had a higher level of hemoglobin, hematocrit, mean corpuscular hemoglobin concentration (MCHC), and the number of monocytes as well as a tendency toward a higher level of mean corpuscular hemoglobin (MCH) and the number of lymphocytes on day 27 (Table 6). On day 35, the two groups were similar regarding hematology (Table 7). The biochemical profile on both day 27 (Table 8) and day 35 (Table 9) was largely similar between FFT and CON. Exceptions were higher alanine aminotransferase (ALT) and lower Mg in FFT on day 27. Finally, there was a tendency toward a lower level of aspartate transaminase (AST) in FFT on day 27.

**Table 6:** Hematological parameters of piglets on day 27 (*n* = CON: 6; FFT: 7).

**Parameter**	**Unit**	**Day**	**CON**	**FFT**	***p-*value**
Leukocytes	bill/L	27	8.62 ± 1.19	9.87 ± 0.96	NS
Erythrocytes	trill/L	27	5.16 ± 0.41	5.65 ± 0.18	0.02^*^
Hemoglobin	mmol/L	27	5.45 ± 0.45	6.79 ± 0.30	0.007
Hematocrit	L/L	27	0.29 ± 0.02	0.34 ± 0.01	0.01^*^
MCH	fmol	27	1.06 ± 0.05	1.20 ± 0.04	0.06
MCHC	mmol/L	27	19 ± 0.32	20 ± 0.20	0.02^*^
Thrombocytes	mia/L	27	625 ± 98	468 ± 54.2	NS
MPV	fL	27	9.48 ± 0.23	9.19 ± 0.33	NS
MCV	fL	27	55.6 ± 2.07	60 ± 1.48	NS
MPC	g/L	27	239 ± 2.36	230 ± 3.89	NS
Lymphocytes	%	27	57.9 ± 6.34	54.1 ± 1.69	NS
Neutrophils	%	27	34.9 ± 6.07	37.4 ± 1.59	NS
Monocytes	%	27	2.28 ± 0.14	2.81 ± 0.35	0.02^*^
Eosinophils	%	27	3.58 ± 0.87	4.93 ± 0.63	NS
Basophils	%	27	0.31 ± 0.03	0.29 ± 0.04	NS
LUC	%	27	1.05 ± 0.23	0.44 ± 0.09	NS
Neutrophils	bill/L	27	3.34 ± 1.17	3.75 ± 0.51	NS
Lymphocytes	bill /L	27	4.66 ± 0.27	5.30 ± 0.45	0.09
Monocytes	bill /L	27	0.20 ± 0.02	0.27 ± 0.03	0.001^*^#
Eosinophils	bill /L	27	0.32 ± 0.09	0.48 ± 0.07	NS
Basophils	bill /L	27	0.03 ± 0.00	0.03 ± 0.00	NS
LUC	bill /L	27	0.09 ± 0.02	0.04 ± 0.01	NS
Reticulocytes	%	27	6.13 ± 1.18	4.19 ± 0.46	0.08
Reticulocytes	bill /L	27	319 ± 72.2	236 ± 25.7	NS

CON, Control, FFT, Fecal filtrate transplantation. MCH, Mean corpuscular hemoglobin, MCHC, Mean corpuscular hemoglobin concentration, MPV, Mean pallet volume, MCV, Mean corpuscular volume, MPC, Mean platelet count, LUC, Large unstained. Values are expressed as mean ± SD. ^*^p < 0.05. NS: Not significant. #: log-transformed data.

**Table 7:** Hematological parameters of piglets on day 35 (*n* = CON: 6; FFT: 8).

**Parameter**	**Unit**	**Day**	**CON**	**FFT**	***p-*value**
Leukocytes	bill /L	35	16.3 ± 1.63	12.4 ± 1.91	NS
Erythrocytes	trill/L	35	6.16 ± 0.29	6.31 ± 0.15	NS
Hemoglobin	mmol/L	35	6.72 ± 0.48	7.05 ± 0.21	NS
Hematocrit	L/L	35	0.33 ± 0.02	0.34 ± 0.01	NS
MCH	fmol	35	1.09 ± 0.06	1.12 ±0.02	NS
MCHC	mmol/L	35	20.4 ± 0.38	20.6 ± 0.22	NS
Thrombocytes	mia/L	35	436 ± 59.9	493 ± 42.1	NS
MPV	fL	35	9.38 ± 0.30	9.05 ± 0.12	NS
MCV	fL	35	53.3 ± 1.85	54.2 ± 0.63	NS
MPC	g/L	35	237 ± 4.54	235 ± 2.44	NS
Lymphocytes	%	35	39.3 ± 4.12	45.2 ± 4.90	NS
Neutrophils	%	35	55.4 ± 3.89	50.0 ± 4.36	NS
Monocytes	%	35	1.83 ± 0.21	0.61 ± 0.22	NS
Eosinophils	%	35	2.90 ± 0.98	2.25 ± 0.98	NS
Basophils	%	35	0.23 ± 0.03	0.25 ± 0.02	NS
LUC	%	35	0.37 ± 0.04	0.31 ± 0.04	NS
Neutrophils	bill /L	35	9.31 ± 1.54	6.42 ± 1.5	NS
Lymphocytes	bill /L	35	6.08 ± 0.34	5.30 ± 0.49	NS
Monocytes	bill /L	35	0.30 ± 0.05	0.24 ± 0.02	NS
Eosinophils	bill /L	35	0.49 ± 0.18	0.34 ± 0.19	NS
Basophils	bill /L	35	0.04 ± 0.01	0.03 ± 0.00	NS
LUC	bill /L	35	0.06 ± 0.01	0.04 ± 0.01	NS
Reticulocytes	%	35	1.18 ± 0.49	0.98 ± 0.20	NS
Reticulocytes	bill /L	35	71.1 ± 25.6	61.9 ± 12.8	NS

CON, Control, FFT, Fecal filtrate transplantation. MCH, Mean corpuscular hemoglobin, MCHC, Mean corpuscular hemoglobin concentration, MPV, Mean pallet volume, MCV, Mean corpuscular volume, MPC, Mean platelet count, LUC, Large unstained. Values are expressed as mean ± SD. NS: Not significant. LUC: Large unstained cells.

**Table 8:** Biochemistry parameters of piglets on day 27 (*n* = CON: 6; FFT: 7).

**Parameter**	**Unit**	**Day**	**CON**	**FFT**	***p-*value**
Albumin	g/L	27	28.5 ± 4.74	26.1 ± 5.63	NS
Total protein	g/L	27	43.3 ± 8.54	39.5 ± 7.87	NS
BASP	U/L	27	774 ± 119	656 ± 191	NS
ALT	U/L	27	29.3 ± 4.03	44.4 ± 5.25	0.003^*^
Total bilirubin	Umol/L	27	3 ± 0.89	3.86 ± 2.67	NS
Creatinine kinase	U/L	27	359 ± 136	379 ± 150	NS
Cholesterol	mmol/L	27	3.27 ± 0.60	3.15 ± 0.41	NS
Creatinine	umol/L	27	69.8 ± 13.2	70.6 ± 10.9	NS
Iron	umol/L	27	15.2 ± 15	19.6 ± 9.57	NS
IP	mmol/L	27	2.82 ± 0.49	2.57 ± 0.41	NS
AST	U/L	27	50.0 ± 18.7	39.0 ± 11.7	0.08
Urea nitrogen	mmol/L	27	3.92 ± 1.37	3.36 ± 0.93	NS
GGT	U/L	27	15.8 ± 4.26	15.0 ± 8.21	NS
CA	mmol/L	27	1.85 ± 0.24	1.74 ± 0.31	NS
MG	mmol/L	27	0.99 ± 0.13	0.87 ± 0.12	0.03^*^
NA	mmol/L	27	136 ± 17.2	129 ± 15.1	NS
K	mmol/L	27	3.98 ± 0.66	3.15 ± 0.48	NS
Triglycerides	mmol/L	27	0.63 ± 0.25	0.75 ± 0.35	NS

CON, Control, FFT, Fecal filtrate transplantation. BASP, Bromsulphthalein, ALT, Alanine aminotransferase, IP, Immunoprecipitation, AST, Aspartate aminotransferase, GGT, Gamma-glutamyl transferase. Values are expressed as mean ± SD. ^*^p < 0.05. NS: Not significant.

**Table 9:** Biochemistry parameters of piglets on day 35 (*n* = CON: 6; FFT: 8).

**Parameter**	**Unit**	**Day**	**CON**	**FFT**	***p-*value**
Albumin	g/L	35	30.6 ± 7.0	32.1 ± 6.0	NS
Total protein	g/L	35	48.7 ± 12.2	46.9 ± 12.3	NS
ALP	U/L	35	157 ± 213	184 ± 166	NS
ALT	U/L	35	45.0 ± 4.44	40.0 ± 4.80	NS
Total bilirubin	Umol/L	35	0.33 ± 0.82	0.25 ± 0.46	NS
Creatinine kinase	U/L	35	1,067 ± 1,947	222 ± 68.4	NS
Cholesterol	mmol/L	35	1.78 ± 0.36	1.73 ± 0.32	NS
Creatinine	umol/L	35	82.3 ± 24.8	103 ± 25.9	NS
Iron	umol/L	35	8.20 ± 13.5	15.1 ± 17.4	NS
IP	mmol/L	35	2.35 ± 0.62	2.24 ± 0.40	NS
AST	U/L	35	33.8 ± 4.38	30.6 ± 10	NS
Urea nitrogen	mmol/L	35	4.12 ± 1.41	5.34 ± 2.58	NS
GGT	U/L	35	15.2 ± 8.82	14.9 ± 8.53	NS
Ca	mmol/L	35	1.01 ± 1.54	1.56 ± 1.59	NS
Mg	mmol/L	35	0.49 ± 0.49	0.57 ± 0.43	NS
Na	mmol/L	35	139 ± 20.1	139 ± 18.2	NS
K	mmol/L	35	16.4 ± 8.97	13.60 ± 11.1	NS
Triglycerides	mmol/L	35	0.42 ± 0.10	0.35 ± 0.16	NS

CON, Control, FFT, Fecal filtrate transplantation. ALP, Alkaline phosphatase, ALT, Alanine aminotransferase, IP, Inorganic phosphate, AST, Aspartate aminotransferase, GGT, Gamma-glutamyl transferase. Values are expressed as mean ± SD. NS: Not significant.

### 3.2. Gut structural and gut functional evaluation

Gut mucosal percentages were similar between CON and FFT on day 27 (61.8% ± 8.40 vs. 61.4% ± 6.61) and on day 35 (60.9% ± 3.15 vs. 62.7% ± 5.32). Mucosal enzyme activity was also similar between FFT and CON on both days 27 and 35 (Tables 10, 11).

**Table 10:** Gut brush border enzymes of piglets on day 27 (*n* = CON: 6; FFT: 7).

**Parameter**	**Unit**	**Day**	**CON**	**FFT**	***p-*value**
**Disaccharidases**					
Lactase	U/g	27	4.91 ± 5.55	4.03 ± 2.76	NS
Maltase	U/g	27	3.91 ± 2.97	4.80 ± 4.35	NS
Sucrase	U/g	27	0.75 ± 0.75	0.53 ± 0.50	NS
**Peptidases**					
ApN	U/g	27	6.25 ± 4.08	6.41 ± 3.10	NS
ApA	U/g	27	3.66 ± 2.92	2.90 ± 1.55	NS
DPPIV	U/g	27	2.88 ± 1.78	2.76 ± 1.21	NS

CON, Control, FFT, Fecal filtrate transplantation. ApN, Aminopeptidase N, ApA, Aminopeptidase A, DPP IV, Dipeptidyl peptidase 4. Values are expressed as mean ± SD. NS: Not significant.

**Table 11:** Gut brush border enzymes of piglets on day 35 (*n* = CON: 6; FFT: 8).

**Parameter**	**Unit**	**Day**	**CON**	**FFT**	***p-*value**
**Disaccharidases**					
Lactase	U/g	35	0.28 ± 0.22	0.39 ± 0.10	NS
Maltase	U/g	35	4.86 ± 0.22	4.58 ± 1.92	NS
Sucrase	U/g	35	0.73 ± 0.62	0.46 ± 0.24	NS
**Peptidases**					
ApN	U/g	35	3.56 ± 1.19	3.25 ± 0.83	NS
ApA	U/g	35	1.33 ± 0.78	1.22 ± 0.20	NS
DPPIV	U/g	35	1.26 ± 0.50	1.40 ± 0.34	NS

CON, Control, FFT, Fecal filtrate transplantation. ApN, Aminopeptidase N, ApA, Aminopeptidase A, DPP IV, Dipeptidyl peptidase 4. Values are expressed as mean ± SD. NS: Not significant.

### 3.3. Gut microbiota composition

**Figure 3:**
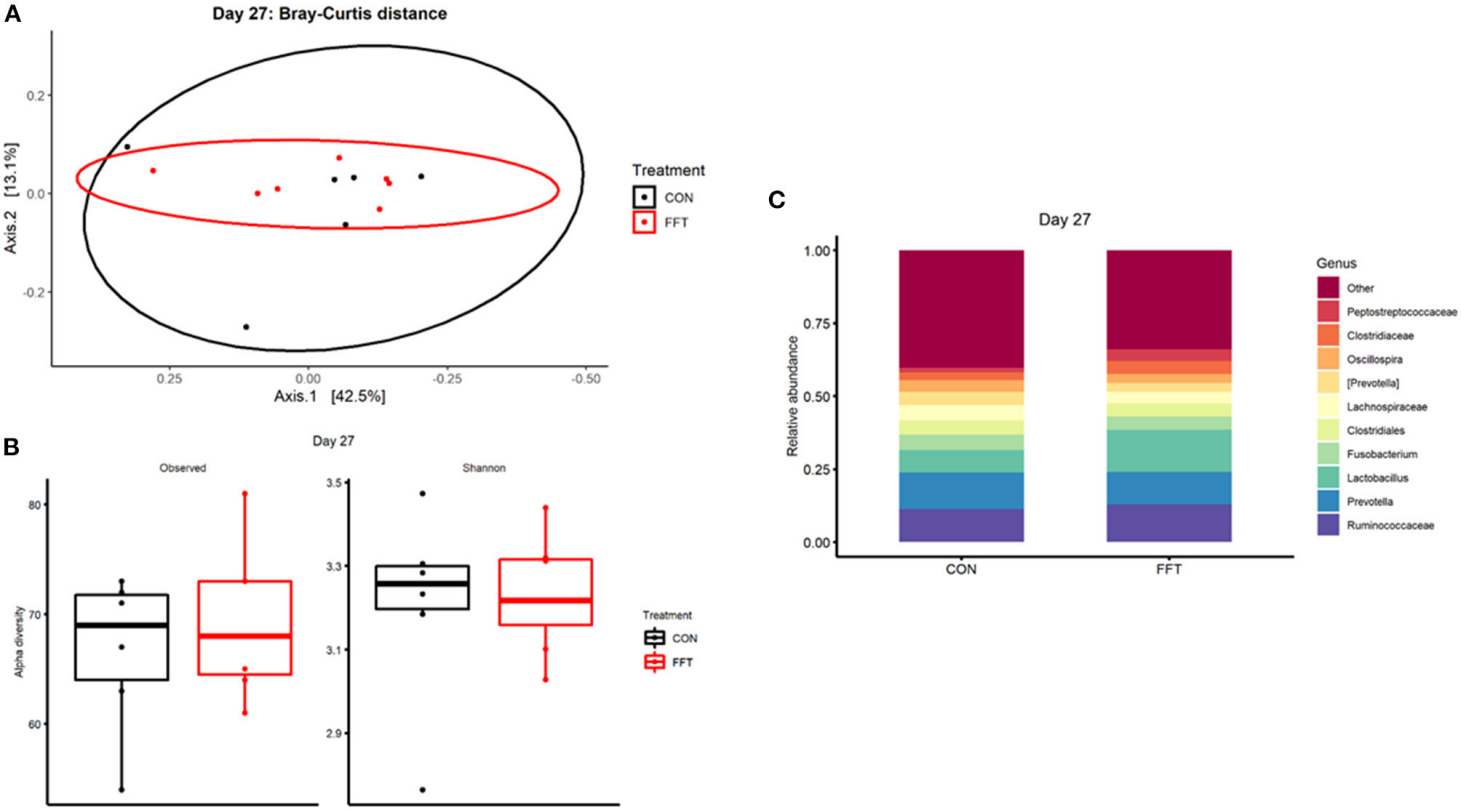
Gut microbiota composition after treatment with either fecal filtrate transplantation (FFT) or sterile saline (CON). **(A)** Unweighted metrics-based measure of beta diversity on day 27. **(B)** Observed and Shannon index as a measure of alpha diversity on day 27. **(C)** Relative bacterial abundances from colon luminal content presented as stacked bar graphs at genus levels on day 27.

**Figure 4:**
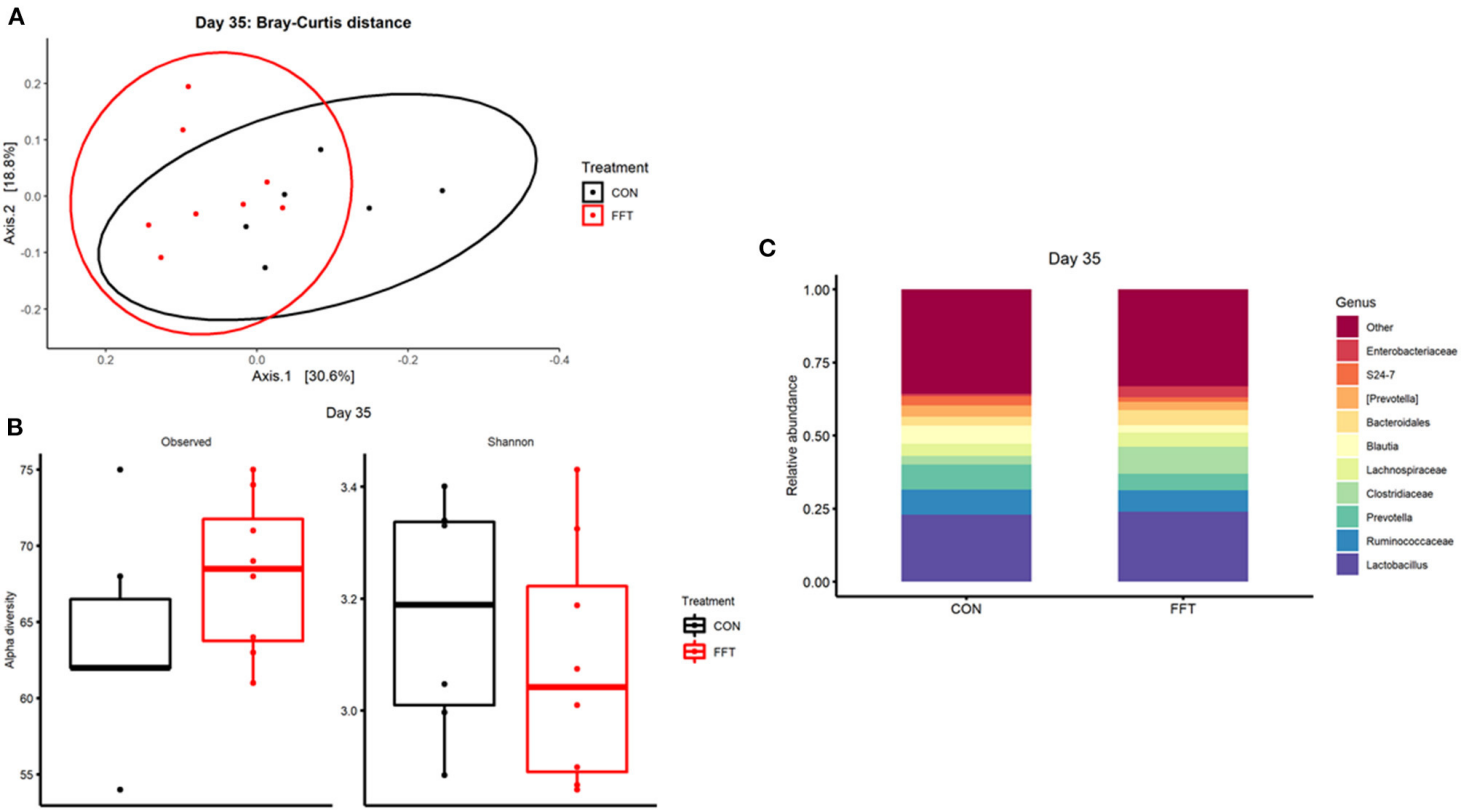
Gut microbiota composition after treatment with either fecal filtrate transplantation (FFT) or sterile saline (CON). **(A)** Unweighted metrics-based measure of beta diversity on day 35. **(B)** Observed and Shannon index as a measure of alpha diversity on day 35. **(C)** Relative bacterial abundances from colon luminal content presented as stacked bar graphs at genus levels on day 35.

We observed only minor effects of the FFT intervention on the recipient gut microbiome community. On day 27, i.e., just prior to weaning, we found similar composition and diversity between the two groups with no differences in relative abundance of single genera (Figure 3). On day 35, we observed a marginal difference in microbiome composition between the groups (Bray-Curtis dissimilarity, PERMANOVA R^2^ = 0.18 and *p* = 0.006, Figure 4). However, this was not accompanied by differences in alpha diversity, whereas differential relative abundances occurred only in a limited number of low-abundant genera (Supplementary Figure 1).

## 4. Discussion

We have shown that early postnatal inoculation of fecal filtrates, reduces PWD. While the effects of FFT have been studied in neonatal pigs (25), the effect in post-weaning pigs has, to the best of our knowledge, not been described before. On the background of the findings in neonatal pigs (32) and in adult humans (26), FFT has already proven to be efficient in treating gastrointestinal infections. From this, we hypothesized that early postnatal FFT would have similar effects on PWD in piglets. Whereas there was only limited diarrhea in the suckling period in any of the two groups, there was a clear difference in the post-weaning period with FFT having little or no diarrhea relative to CON. This suggests that FFT has a positive clinical effect even several weeks after inoculation, and we speculate that the effect is driven by its content of bacteriophages as others have shown positive effects of specific bacteriophages against PWD (27, 33). Similar results have been shown in studies that used early intervention with intact feces (13, 17).

Although we did not design the study to compare fecal filtrates vs. intact feces on the recipient gut microbiome, we have previously found substantial short-term changes to the recipient gut microbiome after FFT in preterm, colostrum-deprived piglets (25). In comparison, the microbiome differences in the current study are minor. One reason may be that the PWD in the CON group was no longer present on the day of sample collection, i.e., day 35. From this notion, the two groups may have converged over time, leaving only marginal differences in the 16s microbiome. It is also plausible that differences cannot be adequately detected at the genus level using the 16s-based profiling of the microbiome. Other analytical approaches like metagenomics, would be required for further in-depth characterization, i.e., including also the population of bacteriophages. In the current study, we speculate that these conventionally reared and adequately immunized animals harbor a more resilient gut microbiome that is less prone to phage predation or prophage integration, which results in negligible changes to the overall gut microbiome. Moreover, we merely analyzed the bacterial composition of the gut luminal compartment, whereas we expect diarrhea-associated pathogens to mainly occupy the mucosal niche. Hence, our data on the gut luminal microbiome might not properly elucidate the potential interaction between phages and bacteria in close proximity to the epithelial lining.

Whereas, growth in the suckling period was similar for the two groups, there was a weight loss in the post-weaning phase, which was surprisingly most pronounced for the FFT group even though they had less diarrhea. We speculate that this results from edema formation in the CON group, but as body composition, including body water percentage, was a defined endpoint, we cannot be conclusive. Further, the current pilot study had a small sample size, and the pre-weaning random selection of pigs from each litter to be tissue-collected at this time point may have induced a slight selection bias. The random selection led to an overrepresentation of large CON pigs to be tissue-collected on day 27, and consequently, the remaining CON pigs that were weaned had lower body weight. Whether this discrepancy in body weight at weaning influenced post-weaning growth remains speculative. Hosseindoust et al. and Kim et al. (34, 35) found that a cocktail of bacteriophages as a supplement in the diet improved overall growth performance in weanling pigs, while, Yan et al. (36) found no effect on ADG in growing pigs after a dietary supplementation with anti-*Salmonella* bacteriophages. These opposing findings may result from a number of reasons including different diet compositions, interactions with feed additives and probiotics, different health statuses, different types and amounts of bacteriophages, volume, and days of administration of bacteriophages.

We found more gastric content in CON vs. FFT in post-weaning pigs. As all pigs had *ad libitum* access to feed during the entire post-weaning period, we speculate that reduced gastric emptying in CON may be a plausible reason. Whereas, Snoeck et al. (37) showed that gastric transit time is short in suckling pigs, it is transiently prolonged in newly weaned pigs and then after 3 weeks returns to levels similar to suckling pigs. Furthermore, parameters such as myoelectric activities, amplitude, and frequency of gastric contractions are all found to be negatively affected by weaning (38). This could contribute to prolonged gastric emptying and overall motility of the stomach. The finding of less gastric content in FFT may thus indicate better gastric emptying as a proxy for better function of the gastrointestinal tract.

At weaning the CON group showed lower levels of monocytes, erythrocytes, lymphocytes, hemoglobin, hematocrit, MCH, and MCHC. These lower levels may either be a result from higher blood volume or reduced hematopoietic bone marrow activity. As the level of total protein in plasma was similar in CON and FFT, the difference in blood cells is unlikely to result from a difference in blood volume. Other plausible reasons could be higher level of inflammation in the CON group or lower hematopoietic activity in the bone marrow.

We assessed mucosal thickness relative to total thickness of the mucosa and submucosa. This indices, together with the indices on mucosal function (i.e., activity of six different digestive enzymes), showed similar values for FFT and CON. Heo et al. (39) state that small intestinal mucosal atrophy after weaning is transient, and that full mucosal thickness is re-stabilized within the first 2 weeks post-weaning. In the current study, it is plausible that post-weaning mucosal regeneration has occurred for all pigs, i.e., explaining why morphology assessments were similar for FFT and CON. It is noteworthy that the weaning transition associates with lowering of the villi and elongation of crypts as a normal physiological transition regardless of whether the intestine is inflamed or healthy. The transition from long slender villi to shorter and more leaf-like villi is presumably a normal adaptation mechanism as the intestine transitions from milk to solid food.

## 5. Conclusion

We have shown that sow feces-derived filtrates provided as oral transplants during the first 6 days of life, reduced PWD, accompanied by a minor modulation of the gut microbiome. Fecal filtrate transplantation did not improve growth or survival. The results suggest that FFT provided in early life can have prophylactic effects several weeks after inoculation, including the weaning transition period.

## Data availability statement

The raw data supporting the conclusions of this article will be made available by the authors, without undue reservation.

## Ethics statement

The animal study was reviewed and approved by Danish Animal Experimentation Inspectorate.

## Author contributions

AB, HS, TT, and CL designed, coordinated, and performed the study. AB and CL participated in analysis and interpretation of the findings. AB, TT, and CL wrote the manuscript. All authors have read and approved the final manuscript.
