# Supplementary material for: Transplantation of fecal filtrate to neonatal pigs reduces post-weaning diarrhea: A pilot study
Source: Front Vet Sci. 2023 Mar 16;10:1110128. doi: 10.3389/fvets.2023.1110128 (PMC10060900; doi:10.3389/fvets.2023.1110128)
Supplement: Supplementary file 1 [file Data_Sheet_1.docx]

Supplementary Material

Transplantation of fecal filtrate to neonatal pigs reduces post-weaning diarrhea, - a pilot study.

Christina Larsen^1^, Amanda B. Andersen^1^, Helena Sato^1^, Anders Brunse^1^, Thomas Thymann^1*^

^1^Department of Veterinary and Animal Science, University of Copenhagen, 68 Dyrlægevej, DK-1870 Frederiksberg C, Denmark

*** Correspondence:**Thomas Thymann, 68 Dyrlægevej, DK-1870 Frederiksberg; phone +45 35332622
[Thomas.thymann@sund.ku.dk](mailto:Thomas.thymann@sund.ku.dk)

# Supplementary Figures and Tables

## Supplementary Figures


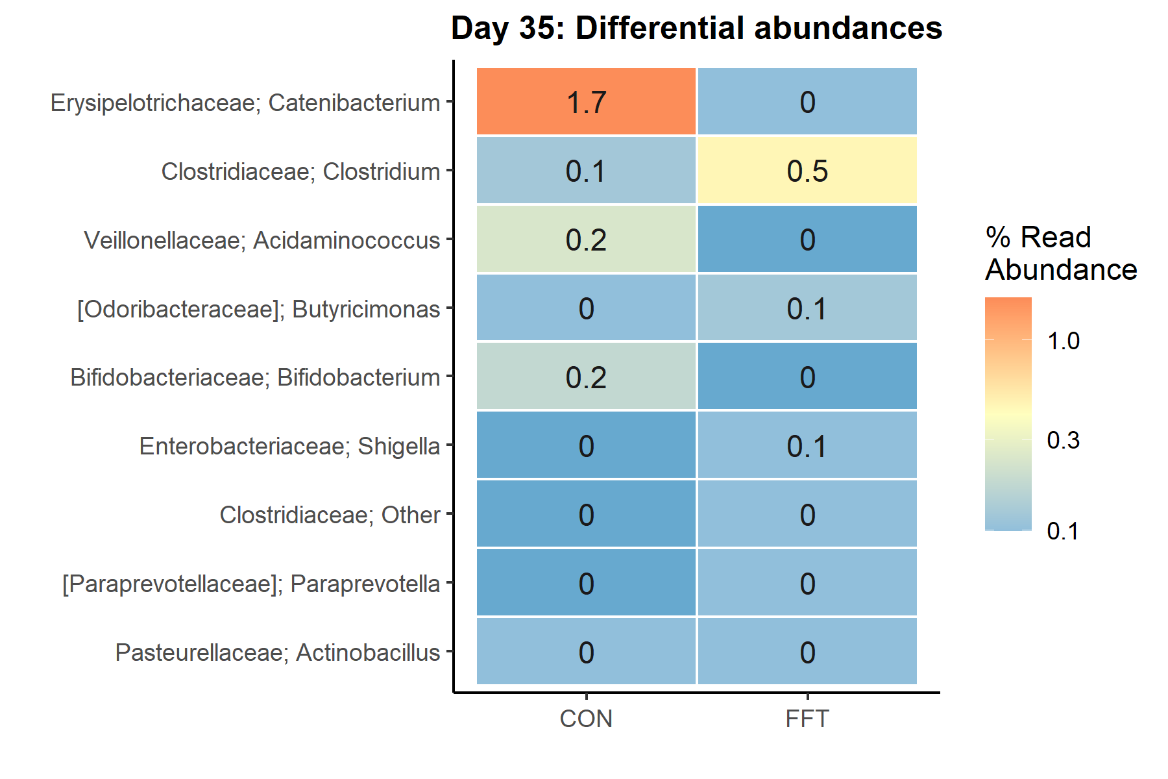


**Supplementary Figure 1.** Heat map of differential abundance on day 35 after treatment with either fecal filtrate transplantation (FFT) or sterile saline (CON).
